# Silica Cladding of Ag Nanoparticles for High Stability and Surface-Enhanced Raman Spectroscopy Performance

**DOI:** 10.1186/s11671-016-1604-5

**Published:** 2016-09-15

**Authors:** Miaomiao Zhao, Hao Guo, Wenyao Liu, Jun Tang, Lei Wang, Binzhen Zhang, Chenyang Xue, Jun Liu, Wendong Zhang

**Affiliations:** Science and Technology on Electronic Test & Measurement Laboratory, North University of China, Taiyuan, Shanxi 030051 China

**Keywords:** SERS, Ag@SiO_2_, Long-term stability, Layer thickness

## Abstract

For high-precision biochemical sensing, surface-enhanced Raman spectroscopy (SERS) has been demonstrated to be a highly sensitive spectroscopic analytical method and Ag is considered to be the best material for SERS performance. Due to the high surface activity of Ag nanoparticles, the high stability of Ag nanostructures, especially in moist environments, is one of the key issues that need to be solved. A method for silica (SiO_2_) cladding of Ag nanoparticles (NPs) is demonstrated here for high sensitivity and long-term stability when putted in aqueous solution. The chemically inert, transparent, hydrophilic, and bio-compatible SiO_2_ surface acts as the protection layer for the Ag nanoparticles, which can also enhance the Raman intensity to a certain extent. In our study, the Ag@SiO_2_ core-shell substrate can detect crystal violet solutions with molar concentrations down to 10^−12^ M. After 24 h of immersion, the reduction in Raman scattering intensity is about 85 % for sole Ag NP films, compared to 12 % for the Ag coated with a 10-nm SiO_2_ layer. This thickness was found to be optimum for Ag@SiO_2_ core-shell substrates with long-term stability and high SERS activity.

**Electronic supplementary material:**

The online version of this article (doi:10.1186/s11671-016-1604-5) contains supplementary material, which is available to authorized users.

## Background

As a powerful spectroscopic technique, surface-enhanced Raman spectroscopy (SERS) has shown promising applications in surface adsorption, biochemical sensing, and trace-level analysis as a result of its high sensitivity, rapid response, and the advantages of nondestructive detection [1–4]. The mechanism for SERS is mainly attributed to the electromagnetic field enhancement caused by the localized surface plasmon resonance of noble metal nanoparticles (NPs). For isolated metal particles, the electromagnetic enhancement can reach up to 10^6^–10^7^, and when in nanogaps (so-called hotspots), it can reach up to 10^10^–10^11^, because of the electromagnetic coupling between the neighboring metal NPs [5, 6].

Among noble metals, Ag is considered to be one of the most promising candidates for SERS applications due to its low loss in optical frequency and high plasmonic efficiency, as well as its lower cost compared to other noble metals [7–10]. However, Ag NPs suffer from sulfur contamination, oxidation, and agglomeration in water and the atmosphere, and the biological incompatibility of Ag is obvious, all of which limit their practical application.

Significant efforts have been devoted to improve the chemical stability of Ag NPs, and core-shell nanostructures are one of the most popular methods, which have been reported in literatures [11, 12]. Ag NPs capped with Au [13], graphene [14], and TiO_2_ [15] have been reported in recent years. Ma et al. [16] prepared ultrathin (~1.5 nm) Al_2_O_3_ films by the atomic layer deposition technique on Ag nanorods that can maintain robust morphologies to a temperature of 400 °C. Li et al. [17] reported the use of a single-atom-thick monolayer of graphene for the protection of Ag NPs that can function as a highly stable SERS substrate for nearly 1 month with ambient aerobic exposure.

The capping thickness of the protection layers can be well controlled by the fabrication technology, which has greatly extended the application of Ag-based SERS substrates in different fields. However, a defect of this coating approach is the tremendous decrease in SERS activity, which is caused by the coating layers that separate the target molecules from the Ag NPs and by the possible morphology changes of the Ag NPs engendered during the coating process. Thus, it is vital to find ways to deposit protective layers which can cap Ag NPs at relatively low temperatures and to precisely control the coating thickness to prohibit the reduction of SERS sensitivity, while still thick enough to be robust towards moist environments.

In this study, Ag NPs were fabricated on 2-in. silicon wafers with a sputtering and vacuum annealing process. We employed inductively coupled plasma-enhanced chemical vapor deposition (ICPECVD) to deposit ultrathin SiO_2_ layers that can cap the exposed surface of Ag NPs with a deposition temperature of 60 °C. After deposition of the SiO_2_ layer, the SERS performance, as well as the coating influences on the stability of the Ag NPs in a water environment, were investigated. It was found that an ultrathin (10 nm) SiO_2_ layer was thick enough to effectively control the distance between the particles to avoid the agglomeration and oxidation even when immersed in water for 15 days. Furthermore, we found that the core-shell structure can improve the SERS performance of Ag substrates when the layer thickness is less than 10 nm.

## Methods

### Fabrication of Ag NP Films

P-type silicon wafers (2 in. in diameter) were used as the substrate. Ag films were deposited in a high vacuum system equipped with a DC magnetron sputtering source (Qprep500, Mantis, UK). The purity of the Ag target was 99.99 %, and DC magnetron sputtering was performed with a DC power of 46 W, an Ar flow rate of 30 sccm and a chamber pressure of 7.5 × 10^−3^ Torr. Then, the sample was annealed at 300 °C for 2 h in a high vacuum system of 5 × 10^−7^ Torr.

### Controllable Growth of Silica Layer

The controllable thickness of the SiO_2_ coating was realized via the ICPECVD system (SI500D, SENTECH, Germany). For the deposition of the SiO_2_ thin films, 130.5 sccm of SiH_4_ and mixtures of 13 sccm of oxygen plus 126 sccm of Ar were introduced to the plasma reactor. The final working pressure during deposition conditions was 1.5 × 10^−2^ Torr, under a deposition temperature of 60 °C. The deposition rate of the SiO_2_ thin film was 20 nm/min on average, and the experimental thickness was measured by a stylus profiler (P-7, KLA-Tencor, USA).

### Morphology and SERS Characterization

The water contact angle against SiO_2_ coating was measured by a contact angle measurement instrument (JGW-360A, Chenghui, China). The morphology of the Ag NPs was characterized by scanning electron microscopy (SUPRA 55 SAPPHIRE, Carl Zeiss AG, Jena, Germany), and the size and density calculations were performed with the assistance of Smile View software.

The stability analysis was conducted by soaking substrates, with and without the cladding layer, into deionized water for 0.5, 1, 5, 10, 24, and 72 h, as well as 15 days. For every period of time, one of the substrates was taken out for testing.

With the distinctive characteristics of Raman peaks, crystal violet (CV) is one of the most commonly used probe molecules in surface-enhanced Raman scattering. In addition, CV is listed as a banned drug of aquaculture by many countries for its high toxicity, high persistence, and cancer-causing peculiarities; thus, it is a good choice for the trace detection experiments [18, 19]. We describe it by the formula of C_25_H_30_ClN_3_, and the relative molecular mass is 407.99. By diluting different masses of solid CV with deionized water, different molar concentrations of CV solution were prepared. All the experiments were performed in a thousand level clean room with a temperature of 20 °C and a relative humidity of 60 %.

SERS characterization was performed after immersion of the samples in CV solutions. The samples were excited using a 514.5-nm laser line from the Raman microscope system (Invia, Renishaw, UK) with an excitation power beam of 5 mW.

## Results and Discussion

### Morphology Characterization of Ag@SiO_2_ Nanostructure

**Fig. 1:**
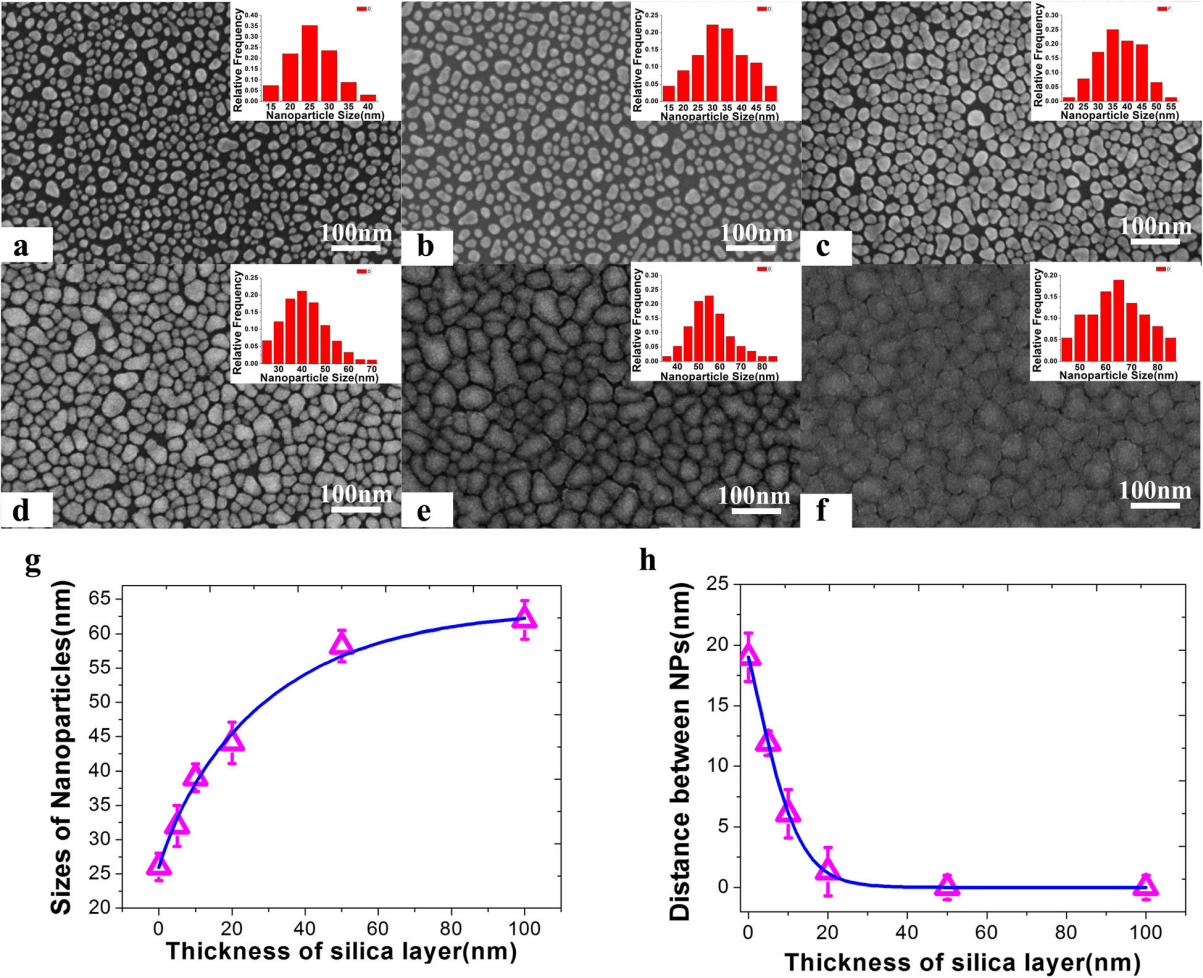
Morphology characterizations of the Ag nanoparticle films coated with different thickness of SiO_2_ layers: **a** 0 nm; **b** 5 nm; **c** 10 nm; **d** 20 nm; **e** 50 nm; **f** 100 nm; **g** variation of nanoparticle diameter as the increase of SiO_2_ thickness; and **h** variation of the distance between Ag NPs as the increase of SiO_2_ thickness

Different film thickness of the SiO_2_ layers can be achieved by controlling the SiO_2_ deposition time, and the morphology of the Ag@SiO_2_ nanostructure was characterized by scanning electron microscopy (SEM), as shown in Fig. 1 and Additional file 1: Figure S1. The corresponding energy spectrum shows the coincident results (Additional file 1: Figure S2). From the characterization results, as shown in Fig. 1g, h, the mean diameters ﻿of Ag@SiO_2_ nanostructures﻿ were 26 ± 2.3, 32 ± 3.6, 39 ± 2, 44.1 ± 3.3, 58.2 ± 2.5, and 62.2 ± 3.2 nm, calculated with the assistance of Smile View software, and the interparticle distance decreased from 19 to 0 nm.

### SERS Characterization of Ag@SiO_2_ Core-Shell Nanostructure and Research on the Enhancement Mechanism

**Fig. 2:**
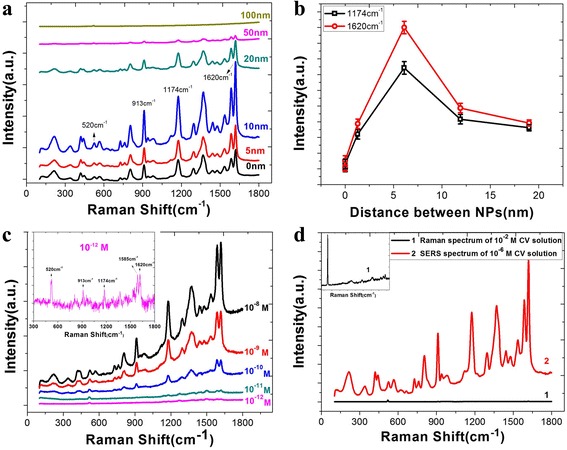
SERS characterizations of Ag@SiO_2_ substrates. **a** SERS spectra of CV on Ag@SiO_2_ substrates with the thickness of SiO_2_ layers vary from 0 to 100 nm. **b** Spectrum intensity calculations of CV at 1174 and 1620 cm^−1^ based on the interparticle distance. **c** SERS spectra of CV absorbed on Ag@SiO_2_ substrate after immersed in different concentrations of CV solution. **d** Normal Raman spectrum of 10^−2^ M CV solution on silicon wafer (*1*) and SERS spectrum of 10^−6^ M CV solution on Ag@SiO_2_ substrate (*2*)

The samples were immersed into 10^−6^ M CV solution, which acted as the probe molecule for 30 min, and then dried in air. The SERS activity of the substrates was tested with the Raman microscope system, and the results are shown in Fig. 2.

There exist various bands for CV at around 913 cm^−1^, attributed to radial aromatic ring skeleton vibrating. The band at 1174 cm^−1^ is related to the C–H bending vibration, while the band at 1620 cm^−1^ is related to the C=C stretching vibration. As shown in Fig. 2a, the thickness of 10 nm acts as the turning point for Raman intensity, and the calculated results reveal that Ag NPs coated with the appropriate shell thickness can improve the sensitivity of SERS active substrates. The highest enhancement in Raman scattering is threefold for the 10-nm shell compared to the uncoated Ag NPs. Besides, the reproducibility of the Ag@SiO_2_ core-shell structure is good by comparing the SERS performance of 12 different 10-nm SiO_2_-coated substrates (Additional file 1: Figure S3).

To further characterize the SERS sensitivity of Ag@SiO_2_ with the SiO_2_ shell of 10 nm, we immersed the substrates in different concentrations of CV solution. As shown in Fig. 2c, we can find that quite high sensitivity was achieved with the Ag@SiO_2_ structure, which can detect CV concentrations down to 10^−12^ M with the signal-to-noise ratio of 6.3 dB. Finally, the enhancement factor (EF) was used to characterize the SERS enhancement of Ag@SiO_2_ nanostructures. It was calculated according to the equation as Leem [20] described:

1 $$ \mathrm{E}\mathrm{F}=\frac{I_{\mathrm{sers}}/{C}_{\mathrm{sers}}}{I_{\mathrm{sol}}/{C}_{\mathrm{sol}}} $$

where *I*_sers_ and *I*_sol_ are the normalized Raman peak intensities of the CV molecules absorbed on the SERS substrate and the reference solution of the selected Raman peak at 1620 cm^−1^, respectively. *C*_sers_ is the molar concentration of the CV on SERS substrates, and *C*_sol_ is the molar concentration of the reference CV solution. According to the results of our experiment, the *I*_sers_ and *I*_sol_ are equal to 90,000 and 136, respectively, and the *C*_sers_ and *C*_sol_ are equal to 10^−6^ and 10^−2^ M, respectively. Therefore, the EF of our Ag@SiO_2_ substrate is 6.6 × 10^6^.

**Fig. 3:**
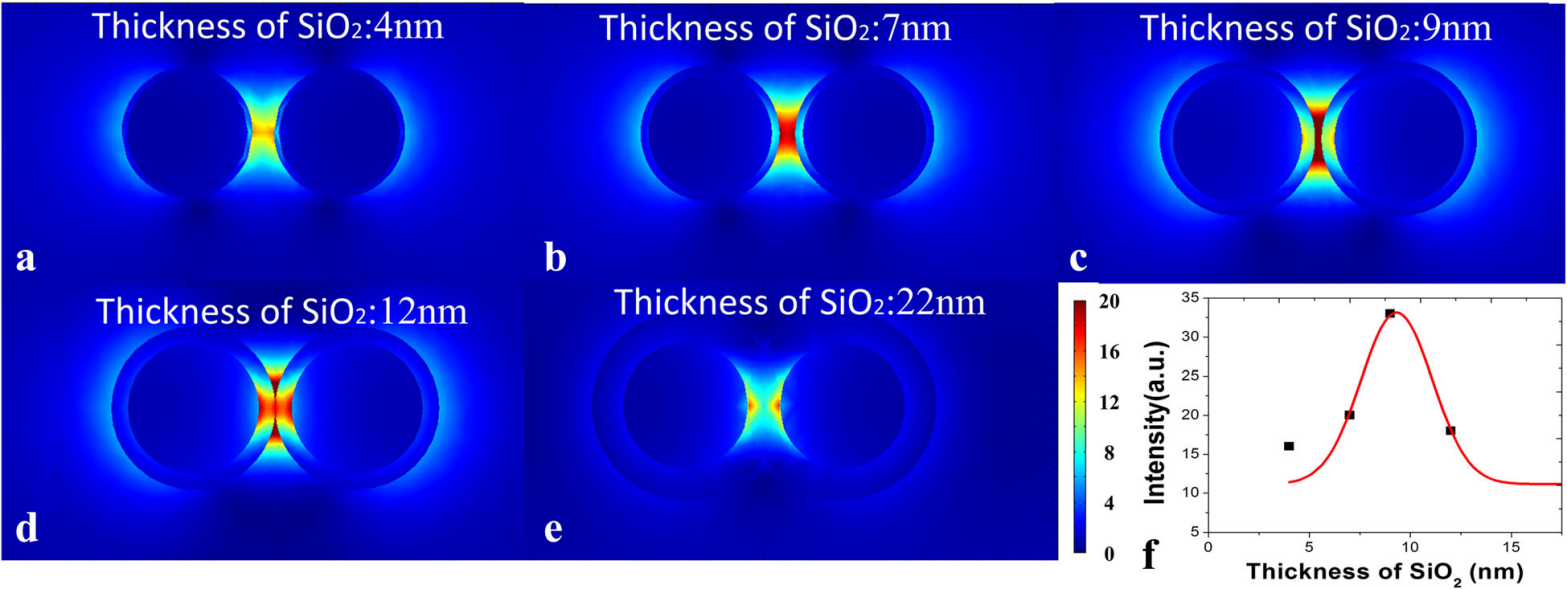
FDTD simulation results for the intensity trend along with the change of spacing. **a** Ag nanoparticles coated with 5 nm SiO_2_, the distance between Ag@SiO_2_ nanostructures is 14 nm. **b** Distance between Ag@SiO_2_ nanostructures is 10 nm. **c** Distance between Ag@SiO_2_ nanostructures is 4 nm. **d** Distance between Ag@SiO_2_ nanostructures is 0 nm. **e** Ag nanoparticles coated with 20 nm SiO_2_; here, we denoted the distance between Ag@SiO_2_ nanostructures as −16 nm. **f** Variation of the SERS intensity along with the spacing between nanoparticles

We believe there were three reasons that a SiO_2_ layer contributes to a higher SERS activity. Firstly, the high refractive index of SiO_2_ layer can confine the light, just like the optical fiber used in the optical communication. Secondly, the multiscattering processes of the light that scattered back and forth at the two curved surfaces of SiO_2_ layers will contribute to a larger enhancement factor in the cavity. Thirdly, the interference of the scattered light from the inner and outer interfaces can diminish the optical field inside the layer, and due to the energy conservation, a relatively larger enhanced field is focused at the outer surface of SiO_2_ layer [21, 22]. Thus, a huge SERS enhancement especially in the cavity between coated Ag NPs can be obtained, which also have been proved by the finite-difference time-domain (FDTD) simulations, as shown in Fig. 3.

In addition, we believe that the high hydrophilic SiO_2_ coating can aggrandize the adsorption quantity of CV molecules on the surface of substrates, which will have an effect on the SERS enhancement to a certain extent [23]. We herein assessed the hydrophilic character with water contact angles. As substantiated by the shift in dynamic contact angle (Additional file 1: Figure S4), a significant increase in surface hydrophilicity of the Ag@SiO_2_ substrates was found. To quantitatively express the impact of the mesoporous structure and hydrophilicity of SiO_2_ layer on the SERS enhancement, we contrasted the Raman intensity of bare Si substrates and Si@SiO_2_ substrate. The enhancement factor here can be calculated according to the Eq. (1) too, and the result is 6.65.

Meanwhile, as a surface-sensitive technique, SERS performance decays exponentially according to the distance between target molecules and SERS substrates. Thus, the increase in SiO_2_ layers separates the probing molecules from Ag NPs, resulting in a sharp reduction in Raman intensity [24].

### Stability Analysis of Ag@SiO_2_ Core-Shell Substrates

**Fig. 4:**
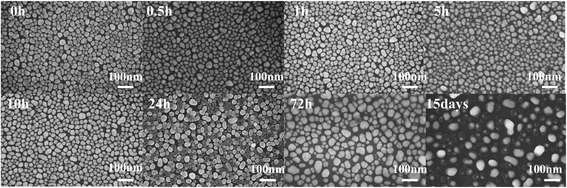
Morphology characterizations of the Ag@SiO_2_ nanostructures after immersing in deionized water for 0, 0.5, 1, 5, 10, 24, and 72 h and 15 days with the thickness of SiO_2_ is 10 nm

To characterize the long-term stability of Ag@SiO_2_ core-shell nanostructures in aqueous solution sensing applications, we put the Ag NP films coated with 0, 10, and 20 nm SiO_2_ layers into deionized water for comparison experiments. At the soaking times of 0.5, 1, 5, 10, 24, and 72 h and 15 days, one of the substrates was taken out and then immersed in a 10^−6^ M CV solution for 10 min. The morphology of the NPs on three kinds of substrates after the immersion process were characterized by SEM. The SEM images (Additional file 1: Figure S5) showed that Ag NP films can be greatly destroyed when exposed to an aqueous solution. After soaking for 0.5 h, agglomeration appeared and almost one third of the NPs were removed, and after 10 h, only a few residues of the Ag NPs can be found which were also strongly agglomerated. Apparently, the stability of Ag NP films is very poor when soaking in the aqueous solution, which can greatly restrict the performance of the SERS activity. Inversely, Ag NPs coated with a 10 nm, as well as a 20-nm SiO_2_ layer, can still remain the morphology even after 24 h of immersion, and the agglomeration of NPs was not discovered after soaking for 15 days. It is obvious that the SiO_2_ layer can protect Ag NPs with a thickness of 10 nm (Fig. 4).

**Fig. 5:**
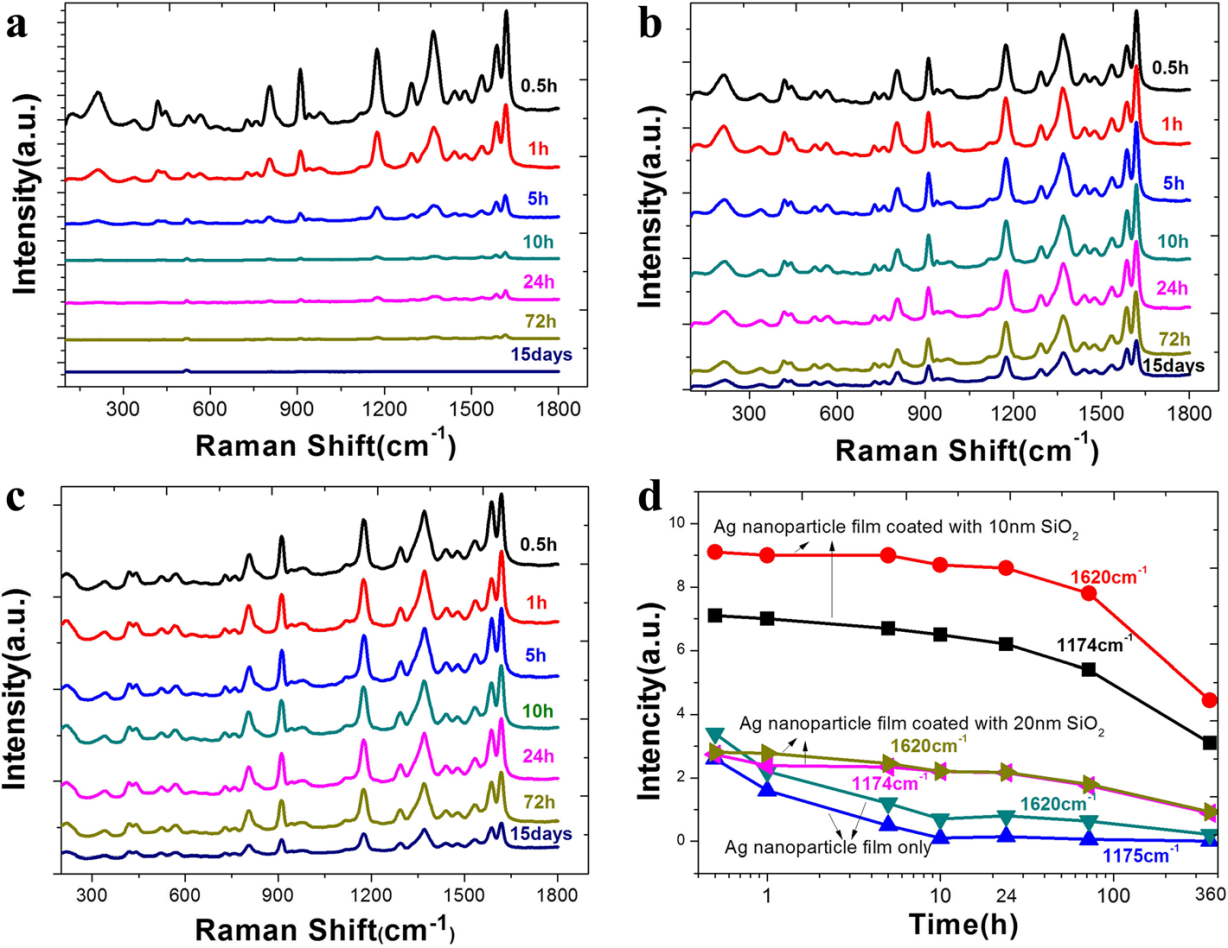
Characterization of SERS-activity, Raman spectra of CV on **a** bare Ag NP films; **b** Ag NP films coated with 10-nm SiO_2_ layer; **c** Ag NP films coated with 20-nm SiO_2_ layer after soaking for different time; and **d** the intensity curves of CV at 1174 and 1620 cm^−1^ absorbed on the substrates with and without SiO_2_

The effect on the SERS activity of substrates during the immersion process was also characterized by Raman microscope system, and the result is shown in Fig. 5. From the figures, we can find that, with the increased soaking time, the Raman intensity of CV absorbed on Ag@SiO_2_ substrates is stable, while for bare Ag NP films, the intensity decreasing sharply. After 24 h of immersion, the decrease in Raman spectrum intensity is about 85 % for bare Ag NP films, compared to 12 % for the Ag NP films with a 10-nm SiO_2_ layer, and 15 % for Ag NP films with a 20-nm SiO_2_ layer (Fig. 5d).

From the results, it can be concluded that SiO_2_-coated metallic NP films can greatly increase the long-term stability compared to bare Ag NP films, especially when the structures are exposed to aqueous solution.

### Applications of Ag@SiO_2_ Core-Shell Substrates

For the aqueous solution sensing applications, Ag@SiO_2_ substrates are further developed with better sensitivity and selectivity through chemical functionalization. We found that a negatively charged surface could be achieved by −COOH functionalization.

Solutions of the food additives brilliant blue (BB) and basic orange (BO) were used. BB molecules in the solution are negatively charged, and BO molecules carry two amino groups. The molarity of BB and BO solutions are 10^−6^ M.

**Fig. 6:**
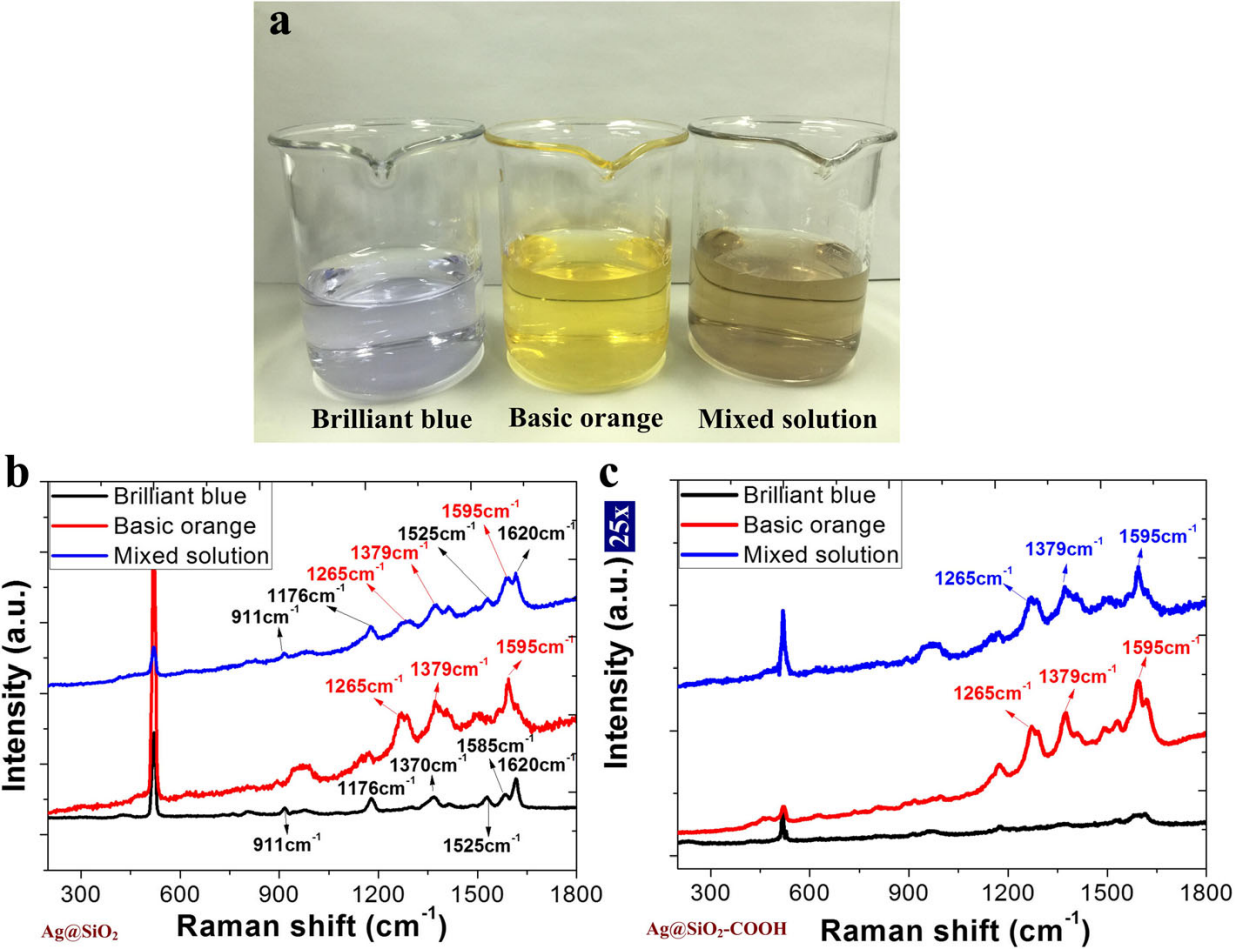
**a** The dilute solutions of brilliant blue (BB), basic orange (BO), and the mixed solution of BB and BO, respectively. **b** The Raman spectra of Ag@SiO_2_ substrate after immersing in the three kinds of solutions. **c** The Raman spectra of Ag@SiO_2_–COOH substrate after immersing in the three kinds of solutions

We immersed the Ag@SiO_2_ as well as the Ag@SiO_2_–COOH substrates in the dilute solutions of BB, BO, and the mixed solution of BB and BO, respectively. The Raman spectra of the three samples are shown in Fig. 6. From Fig. 6b, we can find that both molecules on the nonfunctionalized substrate were detected. Different phenomena were observed with the negatively charged, functionalized substrate (Fig. 6c). In the solutions of BB and the mixed solution of BB and BO, the Raman band due to BB nearly disappeared. This result indicates that the sensitivity to positively charged molecules is improved with the negatively charged SiO_2_ surface and decreased to negatively charged molecules.

The high sensitivity to the specific molecule gives the potential applications of Ag@SiO_2_ in accurate biochemical sensing.

## Conclusions

In this study, a method for SiO_2_ cladding of Ag NPs with long-term stability in aqueous solution was presented and demonstrated. The ICPECVD-grown SiO_2_ layer can suppress the oxidation of Ag NPs effectively and prevent the aggregation and deformation of the particles from occurring. Furthermore, the SERS activity of Ag NP films can be increased by optimizing the thickness of the SiO_2_ dielectric layer that can detect CV concentrations down to 10^−12^ M, and the EF of Ag@SiO_2_ substrates in our experiment can reach up to 6.6 × 10^6^. This work thus provides a straightforward and cost-effective approach to fabricating Ag-based SERS substrates with unprecedented stability and gives us a reliable way to apply Ag nanostructures in the field of solution composition detection. Also, as a bio-compatible material, a SiO_2_ coating of the nanostructure for highly sensitive bio-chemical sensing can be further applied for various biosensor applications.

## Additional file

Additional file 1: Figure S1. The cross-sectional views of Ag@SiO_2_ nanostructures with the thickness of SiO_2_ layers vary from 0 to 50 nm. **Figure S2.** The corresponding energy spectrum of Ag@SiO_2_ nanostructures with the thickness of SiO_2_ layers vary from 0 to 50 nm. **Figure S3.** Characterization of the reproducibility of Ag@SiO_2_ nanostructure (a) Raman spectra of CV on different Ag@SiO_2_ substrates (b). The intensity curves of CV at 1174 and 1620 cm^−1^ absorbed on the substrates. **Figure S4.** A water droplet falling on (a) the Ag film; (b) the Ag@SiO_2_ film; (c) Raman spectra of CV molecules on Si (1)/Si coated with 10-nm SiO_2_ wafer (2). **Figure S5.** Morphology characterizations of the Ag@SiO_2_ nanostructures after immersing in deionized water for 0, 0.5, 1, 5, 10, 24, and 72 h and 15 days with the thicknesses of SiO_2_ are (a) 0 nm (b) 10 nm, and (c) 20 nm. (DOC 19191 kb)
