# Supplementary material for: Silica Cladding of Ag Nanoparticles for High Stability and Surface-Enhanced Raman Spectroscopy Performance
Source: Nanoscale Res Lett. 2016 Sep 15;11:403. doi: 10.1186/s11671-016-1604-5 (PMC5025424; doi:10.1186/s11671-016-1604-5)
Supplement: Additional file 1: Figure S1. — The cross-sectional views of Ag@SiO2 nanostructures with the thickness of SiO2 layers vary from 0 to 50 nm. Figure S2. The corresponding energy spectrum of Ag@SiO2 nanostructures with the thickness of SiO2 layers vary from 0 to 50 nm. Figure S3. Characterization of the reproducibility of Ag@SiO2 nanostructure (a) Raman spectra of CV on different Ag@SiO2 substrates (b). The intensity curves of CV at 1174 and 1620 cm−1 absorbed on the substrates. Figure S4. A water droplet falling on (a) the Ag film; (b) the Ag@SiO2 film; (c) Raman spectra of CV molecules on Si (1)/Si coated with 10-nm SiO2 wafer (2). Figure S5. Morphology characterizations of the Ag@SiO2 nanostructures after immersing in deionized water for 0, 0.5, 1, 5, 10, 24, and 72 h and 15 days with the thicknesses of SiO2 are (a) 0 nm (b) 10 nm, and (c) 20 nm. (DOC 19191 kb) [file 11671_2016_1604_MOESM1_ESM.doc]

**Supplementary material**

After the deposition of SiO_2_ layers, the SEM images as well as the energy spectra of Ag@SiO_2_ nanostructures were characterized by scanning electron microscopy. The cross-sectional views and the energy spectra were shown in Fig. S1 and S2. As shown in Fig. S1, the thickness of SiO_2_ increased, the more concentration of oxygen can be testing in the Ag@SiO_2_ nanostructures.


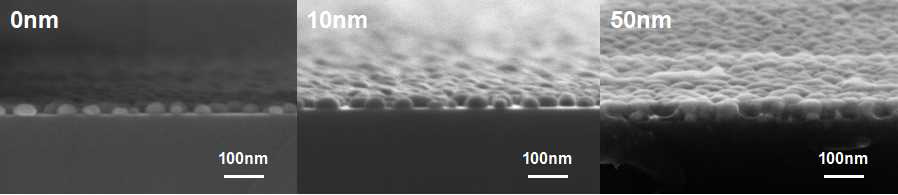


Fig. S1. The cross-sectional views of Ag@SiO_2_ nanostructures with the thickness of SiO_2_ layers vary from 0 nm to 50 nm.

**
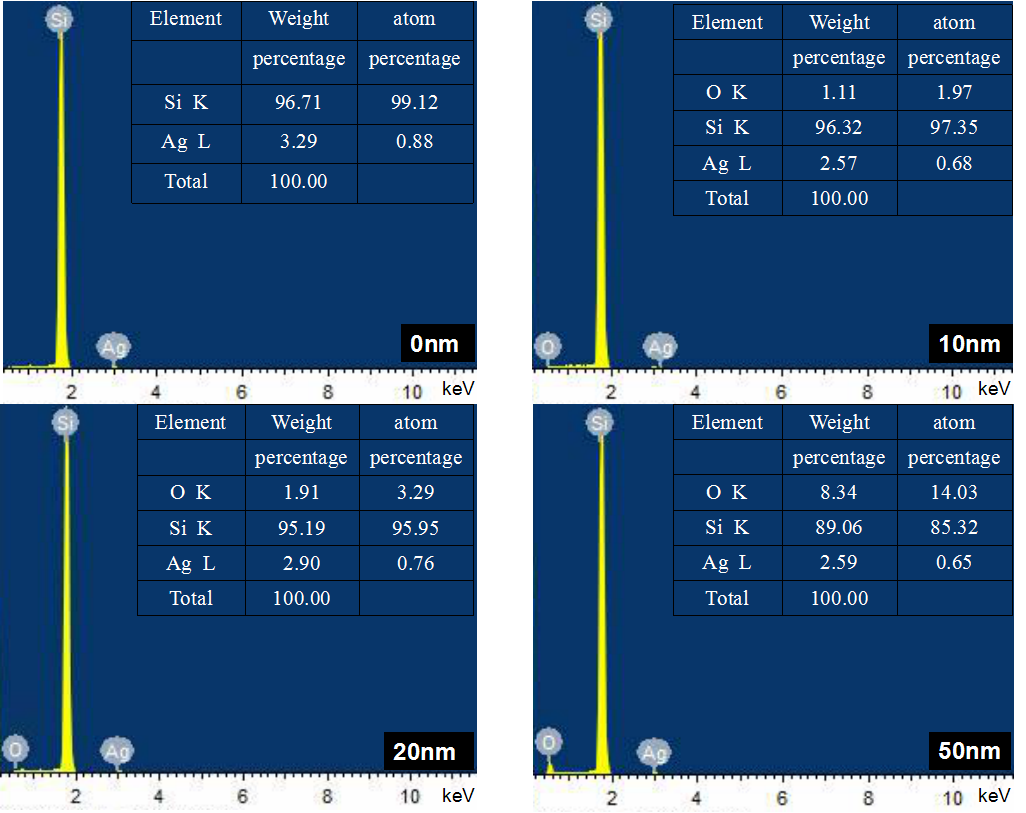
**

Fig. S2. The corresponding energy spectrum of Ag@SiO_2_ nanostructures with the thickness of SiO_2_ layers vary from 0 nm to 50 nm.

To investigate the reproducibility of the Ag@SiO_2_ core-shell structure, we have then compared the SERS performance of twelve different Ag@SiO_2_ substrates, the thickness of SiO_2_ is 10 nm. As it is shown in Fig. S3, the reproducibility is pretty good.


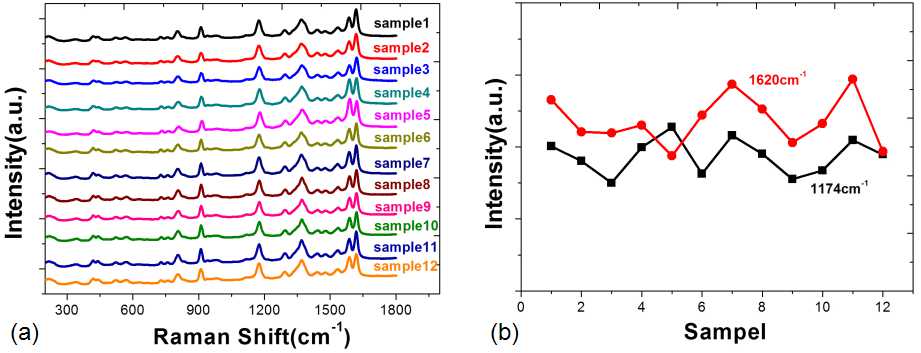


Fig. S3. Characterization of the reproducibility of Ag@SiO_2_ nanostructure (a) Raman spectra of CV on different Ag@SiO_2_ substrates (b) The intensity curves of CV at 1174 cm^–1^ and 1620 cm^–1^ absorbed on the substrates.

The hydrophilic character was assessed here by water contact angles measured with the contact angle measurement instrument by leaving a droplet of water on the surface of the SiO_2_ coating, and the results were shown in Fig. S4. Fig. S4a, b showed the photographs of a water droplet falling on the Ag film coated with and without SiO_2_ coating, the contact angles are 89.18º and 14.58º, respectively. The SiO_2_ coating caused a significant increase in surface hydrophilicity, substantiated by the shift in dynamic contact angle. Then, the Si wafers coated with and without SiO_2_ were used as substrates immersed in 10^–2^ M CV solution for 30 min and flushed with deionized water to ascertain the effect on Raman intensity caused by surface hydrophilicity. Fig. S4c showed the Raman spectra of CV on Si wafer and Si wafer coated with SiO_2_. A higher CV intensity (905 for 1620 cm^−1^) was observed in graph 2 in Fig. S4c, while the other one shows a lower intensity (136 for 1620 cm^−1^) in the same conditions (graph 1). The enhancement factor was calculated according to Eq. (1) and equaled to 6.65.


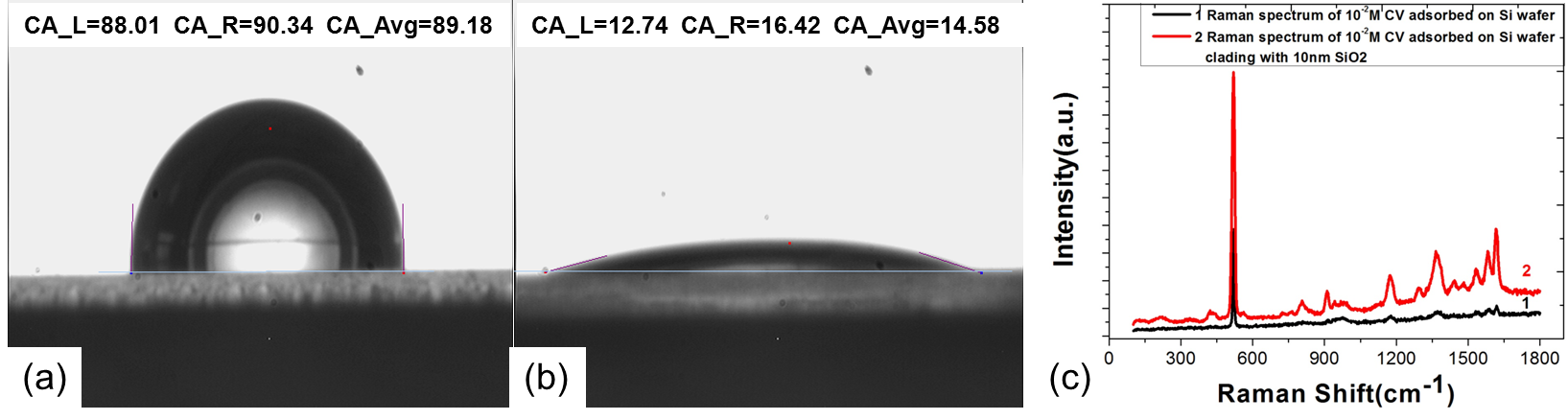


Fig. S4. A water droplet falling on (a) the Ag film; (b) the Ag@SiO_2_ film ; (c) Raman spectra of CV molecules on Si (1) / Si coated with 10nm SiO_2_ wafer (2).

Scanning electron microscopy (sem) images of three kind of substrates texted after immersed in deionized water for 0, 0.5, 1, 5, 10, 24, 72 h and 15 days are shown in Fig. S5. The SEM images show that bare Ag NP films can be greatly destroyed when exposed to an aqueous solution, while Ag NPs coated with a 10 nm, as well as a 20nm SiO_2_ layer, can still remain the morphology even after 24 h of immersion and the agglomeration of NPs was not discovered after soaking for 15 days. It is obvious that the SiO_2_ layer can protect Ag NPs with a thickness of 10 nm.


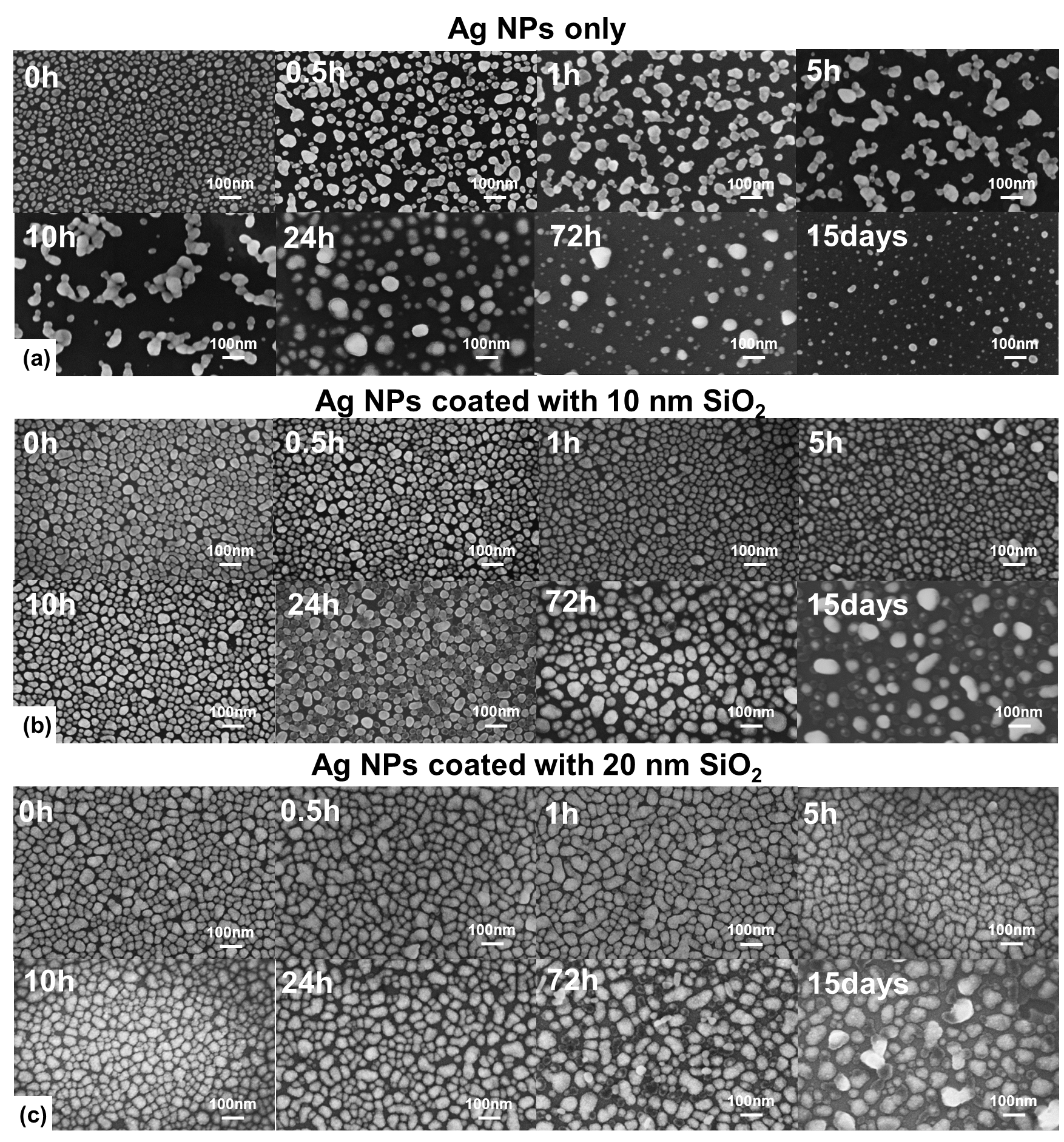


Fig. S5. Morphology characterizations of the Ag@SiO_2_ nanostructures after immersing in deionized water for 0, 0.5, 1, 5, 10, 24, 72h and 15days with the thicknesses of SiO_2_ are (a) 0 nm (b) 10 nm (c) 20 nm.
